# PI3K Signaling in Normal B Cells and Chronic Lymphocytic Leukemia (CLL)

**DOI:** 10.1007/82_2015_484

**Published:** 2015-09-09

**Authors:** Klaus Okkenhaug, Jan A. Burger

**Affiliations:** 16 Laboratory of Lymphocyte Signaling and Development, The Babraham Institute, Cambridge, CB22 3AT UK; 17 Department of Leukemia, MD Anderson Cancer Center, 1515 Holcombe Blvd, Houston, TX 77030 USA

**Keywords:** CLLchronic Lymphocytic Leukemia, Chemokine Receptor, Mantle Cell Lymphoma, CLLchronic Lymphocytic Leukemia Patient, CLLchronic Lymphocytic Leukemia Cell

## Abstract

B cells provide immunity to extracellular pathogens by secreting a diverse repertoire of antibodies with high affinity and specificity for exposed antigens. The B cell receptor (B cell receptor (BCR)) is a transmembrane antibody, which facilitates the clonal selection of B cells producing secreted antibodies of the same specificity. The diverse antibody repertoire is generated by V(D)J recombination of heavy and light chain genes, whereas affinity maturation is mediated by activation-induced cytidine deaminase (AID)-mediated mutagenesis. These processes, which are essential for the generation of adaptive humoral immunity, also render B cells susceptible to chromosomal rearrangements and point mutations that in some cases lead to cancer. In this chapter, we will review the central role of PI3Ks in mediating signals from the B cell receptor that not only facilitate the development of functional B cell repertoire, but also support the growth and survival of neoplastic B cells, focusing on chronic lymphocytic leukemia (chronic lymphocytic leukemia (CLL)) B cells. Perhaps because of the central role played by PI3K in BCR signaling, B cell Leukemia and Lymphomas are the first diseases for which a PI3K inhibitor has been approved for clinical use.

## PI3K Family

The PI3Ks are an ancient family of intracellular kinases that initially evolved to mediate nutrient sensing and metabolic control. In mammals, there are 8 different PI3K catalytic subunits, divided into three classes. Class I PI3Ks phosphorylate phosphatidylinositol(4,5)P_2_ (PIP_2_) to generate phosphatidylinositol (3,4,5)P_3_ (PIP_3_) which acts as pivotal second messenger signaling molecule. In B cells, both Akt and Btk can bind to PIP_3_ via their PH domains. PIP_3_ is essential for the activation of Akt and contributes to the activation of Btk. Less is known about the role of the classes II and III PI3Ks in B cells (Hawkins and Stephens 2015; Okkenhaug 2013b).

**Fig. 1:**
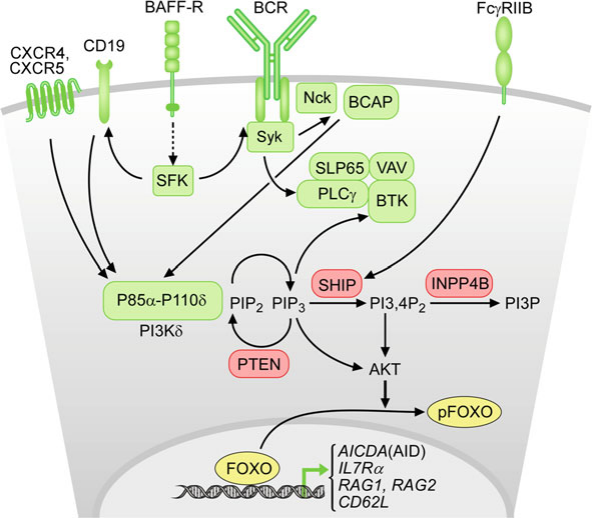
*Activation of PI3K in B cells*. PI3Kδ is a central integrator of signals from the BCR, CD19, CXCR4, CXCR5, and BAFF in B cells. The precise mechanisms linking chemokine and FAFF receptors to PI3K signaling have yet to be fully elucidated. PI3K signaling is antagonized by PTEN and by SHIP which associates with the negative regulator FcγRIIB. PIP_3_ and its metabolite PI(3,4)P_2_ can both activate Akt and its downstream effector, including the inactivation of FOXO. By contrast, BTK can only bind PIP_3_. Not shown in this figure is the complex cross regulation between PI3K and mTOR which is described in the main text and elsewhere

Mammals have 4 different class I PI3Ks. Heterodimers of a regulatory subunit (p85α, p55α, p50α, p85β, or p55γ, collectively referred to as p85) and a catalytic subunit (p110α, p110β, or p110δ) form PI3Kα, PI3Kβ, or PI3Kδ whereas PI3Kγ is a heterodimer of p101 or p84 with p110γ. The p85 regulatory subunits contain SH_2_ domains that recruit PI3K to tyrosine-kinase-linked receptors and their substrates. The p101 and p84 regulatory subunits bind Gβγ subunits released upon G-protein-coupled receptor activation. PI3Kβ can be recruited to tyrosine phosphorylated proteins either via its associated p85 subunit or by direct interaction with Gβγ subunits which bind a unique sequence within the p110 protein (Dbouk et al. 2012). B cells express high levels of PI3Kδ, low levels of PI3Kα and PI3Kγ, and almost no PI3Kβ. PI3Kα and PI3Kδ act redundantly during early B cell development in the bone marrow, whereas PI3Kδ is dominant in mature B cells (Ramadani et al. 2010). PI3K signaling is antagonized by the lipid phosphatases Pten and Ship, which remove the 3 and 5 phosphates from PIP_3_, respectively, and act together to prevent PI3K-dependent B cell transformation (Miletic et al. 2010) (Fig. 1).

## Mechanism of PI3K Signaling in B Cells

### Activation of PI3K by the BCR and CD19

In mature B cells, PI3Kδ is chiefly responsible for PIP_3_ generation and Akt activation (Bilancio et al. 2006; Clayton et al. 2002; Okkenhaug et al. 2002). The best characterized mechanism for regulation of PI3K in B cells involves phosphorylation of the receptor CD19 within two YxxM motifs that bind the p85 SH2 domains with high affinity (Tuveson et al. 1993; Wang et al. 2002) (Fig. 1). CD19 lacks intrinsic or associated tyrosine kinase activity; instead, tyrosine kinases associated with the B cell receptor are mainly responsible for CD19 activation (Buhl and Cambier 1999). Hence, the BCR activates CD19 in trans to recruit PI3K. Consistent with this finding, there are many similarities between mice lacking CD19 and mice lacking p110δ expression in B cells, such as the lack of marginal zone B cells and impaired T-cell-independent immune responses. The membrane-associated protein BCAP can also bind and recruit PI3K via p85, but is not essential for PI3K activity in mature B cells (Yamazaki et al. 2002). Rather, there appears to be redundancy between CD19 and BCAP during early B cell development (Aiba et al. 2008). The BCR is coupled to BCAP via the adapter protein Nck (Castello et al. 2013).

### Atypical Activation of PI3Kδ by Chemokine Receptors in B Cells

B cells are unusual in that PI3Kδ is also the dominant isoform activated by G-protein-coupled receptors (GPCRs) such as the chemokine receptor CXCR5 (Reif et al. 2004). In other cell types, chemokine receptors typically activate PI3Kγ (Andrews et al. 2007). The mechanism underpinning this unusual coupling in B cells remains undefined, but is in part also shared with NK cells (Saudemont et al. 2009). As a consequence, PI3Kγ-deficient B cells are phenotypically normal.

### BAFF Signals via PI3K to Promote B Cell Survival

 BAFF provides an essential survival signal for immature and mature splenic B cells. The BAFF receptor is a member of the TNFR superfamily, which contributes to the activation of NF-κB via the TRAF3-NIK IKK pathway (Mackay et al. 2010; Rickert et al. 2011). Surprisingly, however, deletion of IKK in B cells did not mimic all the severe loss of mature B cells observed in BAFF-R-deficient mice (Jellusova et al. 2013). However, BAFF-dependent cell survival in PI3Kδ null B cells was impaired (Henley et al. 2008), and loss of Pten was sufficient to rescue B cell survival in a BAFF-deficient background (Jellusova et al. 2013). BAFF stimulation results in Akt phosphorylation, and p110δ can be found associate with the BAFF-R (Patke et al. 2006). However, maximal Akt phosphorylation in response to BAFF stimulation was delayed and suggested it may occur downstream of NF-κB activation as Akt phosphorylation after 24 h of stimulation with BAFF was defective in Ikk1^−/−^ B cells (Otipoby et al. 2008). By contrast, Schweighofer and colleagues noted that in Syk-deficient B cells, BAFF-R failed to promote survival and proposed a model whereby BAFF-R engages to BCR signaling complex to activate PI3K since Pten deletion rescued survival signals in B cells lacking Syk (Schweighoffer et al. 2013). Similar results were reported by Hobeika and colleagues; however, the latter study provided evidence also for BAFF-dependent, but Syk-independent survival pathway involving CD19 and PI3K (Hobeika et al. 2015). These results may be reconciled by a model in which BAFF stimulates the activity of a BCR-associated Src-family kinase (SFK) which can phosphorylate the BCR-associated Igα and Igβ, leading to the recruitment of Syk which phosphorylates Bcap to recruit PI3K (Fig. 1). SFK can also phosphorylate CD19 leading to PI3K recruitment in a Syk-independent manner. The relative role of NF-κB versus the PI3K pathway in BAFF-R-dependent survival is likely to depend on the stage of B cell development. Importantly, the transformation of Pten^−/−^Ship^−/−^ B cells was independent of BAFF-R (Jellusova et al. 2013). This last result suggests that blocking BAFF alone is not going to be an effective strategy to treat PI3K-driven B cell leukemia.

### Pdk1, Akt, and Btk Are PIP_3_ Binding Proteins in B Cells

Akt is a Ser/Thr kinase which is absolutely dependent on PI3K for its full activation. Once recruited to a pool of PIP_3_ at the plasma membrane, Akt becomes phosphorylated on Thr308 by Pdk1 which is similarly bound by PIP_3_, but which in contrast to Akt can also be activated to phosphorylate other substrate in the absence of PIP_3_ (Pearce et al. 2010). A major role for Akt in B cells is to phosphorylate and inactivate Foxo transcription factors (Amin and Schlissel 2008; Dengler et al. 2008). Foxo binds to the promoter for the IL7R, Rag1 and Rag2, Aicda, and other key regulators of B cell development and differentiation (Alkhatib et al. 2012; Amin and Schlissel 2008; Dengler et al. 2008). Akt can also contribute to the activation of mechanistic target of rapamycin (mTOR). mTOR exists in two mutually exclusive protein complexes referred to as mTORc1 and mTORc2. Of these, only mTORc1 is inhibited by rapamycin. Akt phosphorylates and inactivates the tuberin proteins Tsc2 and Tsc1, which in turn negatively regulate mTORc1 activity by suppressing Rheb (Limon and Fruman 2012). How mTORc2 is activated remains unknown, but mTORc2 phosphorylates Akt on Ser473, resulting in its full activation. There is hence a circular dependency between Akt and mTOR (Okkenhaug 2013b).

The PH domain of Btk can also bind PIP_3_ with high selectivity, but in contrast to Akt, it is possible to activate Btk under conditions where PI3K activity has been blocked (Matsuda et al. 2009; Scharenberg and Kinet 1998; Suzuki et al. 2003b). This may be because Btk also has SH2 domain that can recruit Btk to the adapter protein Slp65 (Kurosaki 2002). It is possible that PI3K fine-tunes Btk activity following SH2-mediated recruitment of Btk or that there are particular stimulatory conditions under which Btk is more PIP_3_ dependent than others.

## Role of PI3K in B Cell Development and Function

### PI3Kα and PI3Kδ Act Redundancy in Bone Marrow B Cells

Mice lacking both PI3Kα and PI3Kδ in B cell experience a complete block in B cell development shortly after the expression of the immunoglobulin heavy chain (Ramadani et al. 2010). These data indicate that PI3K is not required for V(D)J recombination per se, but rather in order for the pre-BCR formed by the Igh and surrogate light chains to signal further developmental progression. Individual loss of PI3Kα or PI3Kδ alone had no consequence at this developmental stage. Interestingly, loss of Pten or Foxo also causes a block at this developmental stage, but for different reasons. Pten loss leads to increased PI3K-Akt signaling, which in turn leads to the exclusion of Foxo from the nucleus. Because Foxo regulates both IL7R expression and the expression of Rag genes as well as splicing of the lineage-specifying transcription factor Pax-5, Pten^−/−^ or Foxo^−/−^ cells do not express essential genes required for developmental progression and Ig gene recombination (Alkhatib et al. 2012; Amin and Schlissel 2008; Dengler et al. 2008).

### P110δ is the Main PI3K Isoform in Mature B Cells

In mature B cells, complete ablation of BCR-induced Akt phosphorylation is achieved by inhibiting p85α or p110δ alone, but not by deleting p85β, p110α, p110β, or p110γ (Okkenhaug 2013a; Okkenhaug and Fruman 2010). Hence, it appears that the p85α-p110δ heterodimer is preferentially engaged by the BCR. The reason why PI3Kα is not required for mature B cell activation is unclear, but cannot be explained by loss of expression of the p110α subunit which expressed similar levels in immature and mature B cells. Rather, we have speculated that there is a specific requirement for PI3Kδ during agonist activation of the BCR, but that so-called “tonic signaling” can occur via either PI3Kα or PI3Kδ (Okkenhaug 2013b; Ramadani et al. 2010). Tonic BCR signaling is essential for the survival of mature B cells. Therefore, acute ablation of the BCR leads to B cell death in vivo. However, this death can be prevented by expressing an activated form of p110α in B cells, or by deleting Pten or Foxo1 (Srinivasan et al. 2009). The key role for PI3Kδ in BCR signaling means that T-cell-independent immune responses are strongly attenuated in PI3Kδ-deficient mice (Clayton et al. 2002; Okkenhaug et al. 2002; Rolf et al. 2010). However, T-cell-dependent humoral immune responses are relatively unaffected by the loss of the p110δ subunit selectively in B cells (Rolf et al. 2010). This is in part because PI3Kδ actually antagonizes the signals required for immunoglobulin class switching and affinity maturation and also because signaling via CD40 is independent of PI3Kδ. However, follicular helper T cells do not develop in absence of PI3Kδ. As a consequence, congenital or T-cell-specific loss of PI3Kδ leads to attenuated T-cell-dependent humoral immune responses (Rolf et al. 2010). This raises the important point that some effects observed on B cell function after administration of PI3Kδ inhibitors may be secondary to their effects on T cells.

PI3Kδ may also be required for antigen presentation by B cells (Al-Alwan et al. 2007). In addition, IL-21 was shown to selectively stimulate the expression of the costimulatory receptor CD86 via PI3Kδ (Attridge et al. 2014). The expression of CD86 by B cells is essential for their ability to solicit help as it provides costimulation via CD28 expressed by T cells. It should be noted, however, that PI3Kδ-deficient B cells can still undergo CSR in the absence of intrinsic PI3Kδ activity, and moreover, that unrestrained PI3K signaling in Pten^−/−^ B cells antagonizes CSR (Janas et al. 2008; Omori et al. 2006; Rolf et al. 2010; Suzuki et al. 2003a). Indeed, PI3Kδ inhibition leads to enhanced CSR to the IgE isotype (Zhang et al. 2008, 2012). The precise requirement for PI3K during the GC reaction therefore needs to be fully elucidated, but is likely to depend in part on the costimulatory and cytokine milieu.

### PI3Kδ Regulates Marginal Zone B Cell Development

The development of marginal B cells is entirely dependent on PI3Kδ (Okkenhaug et al. 2002; Ramadani et al. 2010). Marginal zone (MZ) B cells secrete mainly IgM or IgG3 which are thought to be broadly protective against microbial antigens, possibly as a first-line defence. MZ B cells are maintained in the periphery of the B cell follicles in the spleen, and it is possible that PI3Kδ promotes their survival by facilitating adhesion to the matrix in the marginal zone (Durand et al. 2009). Treatment of mice with a PI3Kδ inhibitor leads to a gradual depletion of marginal zone B cells from the marginal zone, but not a complete loss of splenic B cells with a marginal zone like phenotype (Durand et al. 2009). This dependency on PI3Kδ for MZ B cells to be nurtured by their local microenvironment may be shared with CLL B cells described next.

## Chronic Lymphocytic Leukemia

### BCR Signaling in CLL Pathogenesis

CLL is a B cell malignancy characterized by the accumulation of mature, CD5^+^CD23^+^ monoclonal B lymphocytes in the blood, secondary lymphatic tissues, and the bone marrow (Chiorazzi et al. 2005). CLL is the most common type of leukemia in adults in Western societies. Several prognostic markers in CLL, such as somatic mutations in the immunoglobulin (Ig) heavy chain variable gene segments (*IGHV*) (Damle et al. 1999; Hamblin et al. 1999), aberrant expression of ZAP-70 (which may act redundantly with SYK) (Chiorazzi 2012), and upregulation of CCL3 (Sivina et al. 2011), are associated with the function of the BCR, suggesting a relationship between BCR signaling, disease progression, and inferior prognosis. Based on the degree of somatic hypermutation of the *IGHVs*, patients can be classified as “unmutated” (U-CLL), if they have 98 % or more sequence homology with the germline sequence, or as “mutated” (M-CLL) cases, if they have less than 98 % sequence homology (Fais et al. 1998). U-CLL cases have a more aggressive clinical course and shorter survival, whereas M-CLL cases have more indolent disease progression and longer survival (Damle et al. 1999; Hamblin et al. 1999). BCRs in CLL patients characteristically have a bias toward usage of restricted *IGHV* and Ig light chain variable gene κ and λ segments (*IGLVκ/λ*) genes, which differ from those of normal B cells, leading to remarkably similar, “stereotyped” third complementarity-determining region of the heavy chain (HCDR3 s) and somatically mutated IGs (Baliakas et al. 2015; Messmer et al. 2004; Stamatopoulos et al. 2007), suggesting antigen-driven selection and expansion of CLL clones. Moreover, recurrent binding of antigen may foster the selection and expansion of B cells clones during early CLL pathogenesis, even before progression to overt CLL (Chiorazzi 2012; Chiorazzi and Efremov 2013; Stevenson et al. 2011). Gene expression profile (GEP) studies revealed that CLL cells from patients with U-CLL show BCR pathway activation (Rosenwald et al. 2001), and comparative GEP analyses demonstrated that BCR signaling and NF-κB signaling are the most prominent pathways activated in CLL cells isolated from lymphatic tissues (Herishanu et al. 2011), indicating that BCR activation is a key driver for CLL proliferation within disease-characteristic proliferation centers (also called pseudo-follicles) in secondary lymphoid tissues.

Two major mechanisms of BCR activation have been described in CLL: ligand (antigen)-induced and ligand-independent autonomous BCR activation (Burger and Chiorazzi 2013). In contrast, activating BCR pathway mutations which are common in diffuse large B cell lymphoma (DLBCL) generally does not appear to play a role in CLL patients (Philippen et al. 2010), except as a treatment resistance mechanism in patients receiving BCR-signaling-targeted therapy. In CLL patients developing ibrutinib resistance, BTK and PLCγ2 mutations have been linked to drug resistance, causing either ineffective drug binding to its target (C481S mutation of BTK) or autonomous BCR pathway activation due to gain-of-function mutations (R665W and L845F mutations in PLCγ2) (Woyach et al. 2014). BCRs from U-CLL patients are more poly-reactive, whereas BCRs from M-CLL cases are more selective, providing high-affinity antigen binding. U-CLL BCRs can recognize auto-antigens and other environmental or microbial antigens (Borche et al. 1990; Broker et al. 1988; Herve et al. 2005; Sthoeger et al. 1989), such as cytoskeletal non-muscle myosin heavy chain IIA and vimentin, as well as the Fc-tail of IgG (“rheumatoid factors”), ssDNA, or dsDNA, LPS, apoptotic cells, insulin and oxidized LDH (Binder et al. 2010; Borche et al. 1990; Catera et al. 2008; Chu et al. 2010; Herve et al. 2005; Lanemo Myhrinder et al. 2008; Sthoeger et al. 1989). Microbial antigens, such as bacterial and fungal antigens, also can be specifically recognized by CLL BCR. M-CLL patients express IGHV3-7 with short HCDR3 sequences, which display high-affinity binding to β-(1,6)-glucan, a major antigenic determinant of yeasts and filamentous fungi (Hoogeboom et al. 2013). Collectively, these findings indicate that antigen selection and affinity maturation promote the expansion of certain CLL clones via antigen-/pathogen-specific BCR signaling, similar to the role of H. pylori in MALT lymphoma pathogenesis.

In addition, two recent studies demonstrated an additional form of auto-reactive BCR signaling in CLL termed autonomous BCR signaling (Duhren-von Minden et al. 2012; Iacovelli et al. 2015). These data are based on experiments in which CLL BCRs are expressed by retroviral gene transfer into mouse cells that lack endogenous BCRs. These CLL BCRs were found to bind via their HCDR3 to an epitope in the second framework region (FR2) of another antibody, inducing Ca^2+^ signaling. The finding could explain the presence of phosphorylated LYN and SYK seen in CLL cells, although it does not appear to account for clinical differences between M-CLL and U-CLL (Duhren-von Minden et al. 2012) or for the lack of CLL cell proliferation in the absence of external BCR stimulation (Hoogeboom et al. 2013). Binder et al. reported an alternative epitope for BCR self-recognition in CLL, located in the framework region 3 of the variable region of IGH (Binder et al. 2013). Recent mouse model work evaluated these different types of antigen-BCR interactions in the Eμ-TCL1 transgenic mouse model of CLL. First, the authors demonstrated that low-affinity BCR interactions with auto-antigens, such as phosphatidylcholine (PtC), are positively selected (Chen et al. 2013; Iacovelli et al. 2015); secondly, BCR signaling in response to such antigens accelerated CLL development, indicating that BCR signaling triggered by external (auto)antigen increases the aggressiveness of the disease. Finally, ligand-independent autonomous signaling of CLL BCRs likely also contributes to the disease process in this model (Iacovelli et al. 2015).

### Role of Chemokine Signaling in CLL

**Fig. 2:**
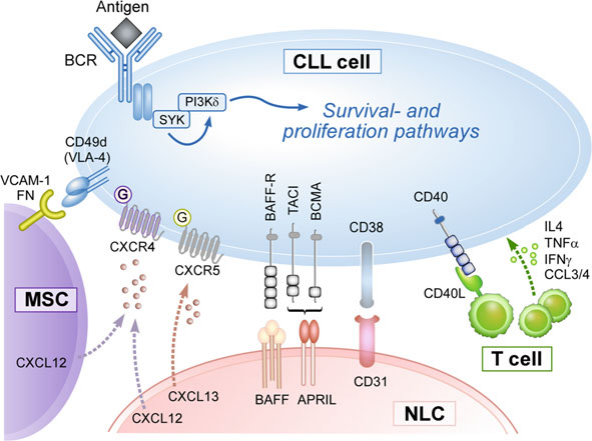
*Cellular and molecular interactions between CLL cells and the tissue microenvironment*. B cell receptor (BCR) signaling is a key pathway for promoting survival and growth of CLL B cells, mediated via upstream kinases SYK and PI3Kδ, and further downstream signaling detailed in Fig. 1. In addition, interactions between CLL cells and T cells are central for the expansion of the malignant CLL clone. Interactions via CD40, expressed on the CLL cells, and CD40 ligand (CD154), as well as cytokines (IL4, TNFα, IFNγ, CCL3, CCL4), play an important role in CLL-T-cell cross talk. CLL cells are attracted and retained in tissue microenvironments, such as the secondary lymphatic tissues and the bone marrow, by chemokines that are constitutively secreted by tissue mesenchymal stromal cells (MSC) and monocyte-derived nurselike cells (NLC). These stromal cells establish chemokine gradients, such as CXCL12 and CXCL13 gradients that attract CLL cells via the corresponding G-protein-coupled chemokine receptors, CXCR4 and CXCR5, respectively. Adhesion molecules on the CLL cells, such as CD49d (VLA-4), cooperate with chemokine receptors during this process. NLC also express BAFF and APRIL, as well as CD31 for activation of respective ligands on the leukemia cells, promoting growth and survival of the CLL cells

Besides its role in BCR signaling, PI3Kδ also plays an important role in CLL cell migration and tissue homing (Fig. 2). Preclinical studies established that PI3Kδ blockade with idelalisib inhibited CLL cell migration in response to tissue homing chemokines (CXCL12 and CXCL13) and impaired leukemia cell adhesion and migration beneath stromal cells that secrete chemokines (Hoellenriegel et al. 2011). These effects are similar to the effects of BTK inhibition on migration and adhesion of normal and malignant B cells (de Rooij et al. 2012; Ponader et al. 2012; Spaargaren et al. 2003) and emphasize that PI3Kδ and BTK share functions in the transmission of signals from chemokine receptors and adhesion molecules. An alternative, not mutually exclusive mechanism for explaining the effects of these kinase inhibitors on B cell trafficking and homing is related directly to the BCR. Activation of the BCR induces inside-out activation of integrins and reorganization of the cytoskeleton (Harwood and Batista 2010), involving small GTPases such as Rac2 and Rap (Arana et al. 2008). Consequently, BCR triggering results in enhanced integrin-mediated B cell adhesion, which, in turn, can be reversed via PI3Kδ inhibition with idelalisib, inhibiting integrin-mediated adhesion of CLL B cells to VCAM1 (CD106) (Fiorcari et al. 2013). These mechanism likely contribute to the “class effect” of PI3Kδ (Brown et al. 2014; Furman et al. 2014), BTK (Byrd et al. 2013), and SYK inhibitors (Friedberg et al. 2010) in CLL and MCL patients (Chang et al. 2013), where a mobilizing effect (“redistribution”) of tissue-resident B cells into the peripheral blood characteristically is seen during the first months of therapy (Burger and Montserrat 2013).

Taking a closer look at the mechanism regulating B cell and CLL cell migration and tissue homing helps us to understand the complex effects of PI3Kδ blockade in CLL. Trafficking of normal lymphocytes, but also of CLL cells between blood and secondary lymphoid tissues, is organized by tissue-specific expression of chemokines and ligand- and activation-regulated expression of chemokine receptors on lymphocytes, cooperating with adhesion molecules and their ligands (Burger and Montserrat 2013; Moser and Loetscher 2001). Lymphocytes in the blood interact with vascular endothelium via adhesion molecules (selectins and integrins) in a process called rolling. Chemokines on the luminal surface of the endothelium activate chemokine receptors on these rolling lymphocytes, which in turn causes integrin activation (Springer 1994), arrest and firm adhesion, followed by transendothelial migration into the tissues, where stromal cells organize the localization and retention of the lymphocytes via chemokine gradients (Campbell et al. 1998). This process, referred to as “tissue homing,” is an integral part of immune surveillance and function of the immune system. Specific to B cells is their localization in germinal centers during adaptive immune responses, where somatic hypermutation and clonal selection occur within distinct regions called dark zone (DZ) and light zone (LZ). PI3Kδ inhibition with idelalisib significantly reduces CLL cell migration in response to CXCL12 and CXCL13, as well as high CXCL13 plasma levels in patients treated with idelalisib (Hoellenriegel et al. 2011).

Normal mechanisms of B cell trafficking and tissue homing are largely preserved in CLL B cells. Blood CLL cells express high levels of CXCR4 (Burger et al. 1999), which are downregulated in tissues by its ligand CXCL12 (Burger et al. 1999). This effect can be used to distinguish proliferating CXCR4^dim^ CLL cells in CXCL12-abundant tissues (bone marrow, lymph nodes) from CXCR4^high^ CLL cells from blood (Bennett et al. 2007; Calissano et al. 2009). CXCR5 is another chemokine receptor expressed at high levels on CLL cells, which controls access of CLL cells to monocyte-derived “nurselike cells” (NLC) (Burkle et al. 2007) and follicular dendritic cells (FDC) (Heinig et al. 2014). In turn, CXCR5-mediated contact to FDC provides proliferative stimuli to the CLL cells (Heinig et al. 2014). CLL cells also can stimulate stromal cells through lymphotoxin-β-receptor activation, resulting in CXCL13 secretion and remodeling of the stromal cell compartment, linking CLL cell homing with shaping of survival niches and access to proliferation stimuli.

Another layer of complexity is added by the fact that activated CLL cells also secrete chemokines (CCL3, CCL4, and CCL22) which further shape the cellular composition of the tissue microenvironment (Burger et al. 2009; Ghia et al. 2002). CCL3 and CCL4 are generally secreted by macrophages, dendritic cells, B and T lymphocytes, and function as chemoattractants for monocytes and lymphocytes (Schall et al. 1993). Previous studies established that CCL3 is a key response gene upregulated in normal and neoplastic B cells in response to BCR signaling (Burger et al. 2009; Eberlein et al. 2010; Herishanu et al. 2011; Krzysiek et al. 1999) and repressed by Bcl-6 (Shaffer et al. 2000). CLL cells upregulate and secrete CCL3 and CCL4 in response to BCR stimulation and in coculture with NLC (Burger et al. 2009), a model system resembling the lymphatic tissue microenvironment (Burger et al. 2000, 2009). High CCL3 plasma concentrations are a robust, independent prognostic marker in CLL (Sivina et al. 2011). This BCR- and NLC-dependent induction of CCL3 and CCL4 is sensitive to inhibition of BCR signaling, using SYK (Hoellenriegel et al. 2012; Quiroga et al. 2009), BTK (Burger et al. 2014; Ponader et al. 2012), or PI3Kδ (Hoellenriegel et al. 2011) inhibitors, both in vitro and in vivo. Based on the postulated function of B-cell-derived CCL3 in normal immune responses, increased CCL3 secretion by CLL cells may cause attraction and homing of accessory cells to the malignant B cells in the tissue microenvironments (Burger et al. 2009; Zucchetto et al. 2009). It is well recognized that CLL cells in the proliferative compartment are interspersed with T cells (Ghia et al. 2002; Patten et al. 2008) and cells of monocyte/macrophage lineage, termed NLC (Burger et al. 2009). Conceivably, CLL cell-derived CCL3 may attract these cells, creating a favorable microenvironment which allows CLL cells to interact with T cells and NLC to receive survival and proliferation signals. This is supported by in vitro (Krzysiek et al. 1999) and in vivo (Bystry et al. 2001; Castellino et al. 2006) studies which indicated that normal B cell activation within lymphoid tissues results in CCL3 and CCL4 secretion, leading to the recruitment of CCR5^+^ regulatory T cells for cognate interactions with B cells and antigen-presenting cells (APCs) (Bystry et al. 2001; Castellino et al. 2006). In ongoing clinical trials with new agents targeting the BCR pathway (SYK, BTK, and PI3Kδ inhibitors), increased levels of CCL3 and CCL4 rapidly normalized after initiation of therapy with the BTK inhibitor ibrutinib (Burger et al. 2014; Ponader et al. 2012) and the PI3Kδ inhibitor idelalisib (Hoellenriegel et al. 2011) (Table 1).

**Table 1:** Selected PI3K, SYK, and BTK inhibitors in B cell malignancies (phase 2 and later, most advanced trial listed)

Disease	Target	Drug	Stage	Study
*CLL/SSL*				
	PI3Kδ	Idelalisib	Approved	NCT01539512
	PI3Kδ/γ	Duvelisib	Phase 3	NCT02004522
	BTK	Ibrutinib	Approved	NCT01578707
iNHL	PI3Kδ	Idelalisib	Approved	NCT01282424
	PI3Kδ/γ	Duvelisib	Phase 2	NCT01882803
DLBCL	SYK	Fostamatinib	Phase 2	NCT01499303
Multiple B cell malignancies	SYK	Entospletinib	Phase 2	NCT01799889
	SYK + PI3Kδ	Entospletinib + idelalisib	Phase 2	NCT01796470

### Role of PI3K in T Cells in CLL

Characterization of preclinical effects of the PI3Kδ inhibitor idelalisib focused on CLL cells (Herman et al. 2010; Hoellenriegel et al. 2011), rather than on other immune cells, such as T cells. However, the idelalisib side effect profile in CLL patients, especially cases of pneumonitis and late-onset diarrhea, points toward other immune-mediated effects (Coutre et al. 2015). T-cell-mediated inflammatory bowel disease seen in the PI3Kδ kinase dead-mouse model may be related to reduced regulatory T cell (T_reg_) function (Okkenhaug et al. 2002; Patton et al. 2006) or increased macrophage response to gut microbiota (Steinbach et al. 2014; Uno et al. 2010). The idelalisib-prescribing information contains a warning for severe diarrhea or colitis, hepatotoxicity, pneumonitis, and intestinal perforation. An early type of diarrhea, which generally occurs within the first 8 weeks, is typically mild and tends to be self-limiting. A second type of diarrhea tends to occur relatively late, is clinically often more complicated, and shows histologic signs of lymphocytic colitis that are reminiscent of those seen in PI3Kδ knockout mice (Coutre et al. 2015; Okkenhaug et al. 2002). Hence, these clinical observations suggest that PI3Kδ inhibition in CLL patients has effects on T cell subsets, triggering inflammatory reactions in a subset of patients. On the other hand, it is tempting to speculate that some of the beneficial clinical activity of idelalisib in CLL may be related to breaking T_reg_-cell-mediated immune tolerance to the CLL cells, as recently described in mouse models of solid tumors (Ali et al. 2014). In addition, there is evidence that Th1 cells can support CLL activation and proliferation (Burgler et al. 2015; Os et al. 2013) and PI3Kδ inhibition can suppress Th1 responses (Okkenhaug et al. 2006; Soond et al. 2010). None of these issues have yet been experimentally addressed in the context of CLL, and therefore, studies of the effects of idelalisib on T cell subsets in CLL patients should become an important part of future correlative studies.

### Targeting BTK and PI3Kδ: Redundant or Synergistic?

Effects of BTK and PI3Kδ inhibition on BCR signaling and B cell migration and adhesion are similar in terms of their effects on calcium signaling and gene regulation (Fruman et al. 2000); however, the kinases diverge at the level of Akt and Foxo which are primarily regulated by PI3K (Fig. 1). Hence, the phenotype and functional defects within the B cell compartment of BTK- or PI3Kδ-deficient mice are similar, but with some key differences (Okkenhaug et al. 2002; Ponader and Burger 2014; Suzuki et al. 2003b). Idelalisib and ibrutinib have multiple similarities in terms of anti-CLL activity. First, they both induce rapid and robust reduction in lymphadenopathy, together with a transient redistribution of CLL cells into the peripheral blood, which over time, while patients are receiving continuous kinase inhibitor therapy, improves and resolves in many patients, especially when given together with other agents, such as anti-CD20 mAbs (Burger et al. 2014; Furman et al. 2014). These clinical responses are thought to be due to dual effects of these kinase inhibitors on migration and tissue homing of the CLL cells on the one hand, and on proliferation and survival of the leukemia cells on the other hand. These similarities between BTK and PI3Kδ inhibition raise the question whether combinations of drugs that target these enzymes, such as idelalisib and ibrutinib, would have any benefit or be redundant. Besides the obvious disadvantage of high treatment costs, such combinations likely have off target effects on the immune system in general, and on T cell in particular that currently are difficult to predict and that could be either beneficial or toxic.

At this time, treatment with these kinase inhibitors is continuous and indefinite in patients who benefit with sustained responses, although a subset of patients treated with ibrutinib develop specific resistance and may benefit from switching to idelalisib. Whether similar resistance to idelalisib will develop, prompting a switch to ibrutinib, remains to be seen. The adverse effect profile or idelalisib and ibrutinib are also different. While a significant proportion of patients on idelalisib develop colitis (Coutre et al. 2015), some ibrutinib patients develop atrial fibrillation (Byrd et al. 2014). With time, it may become possible to stratify patients who are more likely to benefit from BTK or PI3Kδ inhibitors. It also remains possible that next-generation inhibitors will be associated with fewer side effects.

The fact that BTK and PI3Kδ inhibitions typically induce partial remissions with substantial residual disease (Byrd et al. 2013; Furman et al. 2014), even after years of continuous therapy (Byrd et al. 2015), suggests that additional therapeutic intervention, such as immune-mediated therapy, is necessary for disease eradication that would allow therapy discontinuation. Immune-modulatory effects of ibrutinib (Sagiv-Barfi et al. 2015) and PI3Kδ inhibition (Ali et al. 2014), resulting in restoration of cytotoxic T cell function and T-cell-mediated tumor regression, are the most recent exciting effects of this class of agents. Ibrutinib’s T cell modulatory effects are mediated through a shift in the balance between Th1 and Th2 T cells by inhibition of ITK, an essential enzyme in Th2 T cells (Dubovsky et al. 2013; Sagiv-Barfi et al. 2015). PI3Kδ inhibition, on the other hand, disables regulatory T cells and thereby unleashes CD8-positive cytotoxic T cells (Ali et al. 2014). These immune-modulatory effects could support each other when combined and could result in deeper remissions or potentially even in immune-mediated disease eradication. Carefully designed trials with correlative studies that address these mechanistic questions will help to evaluate the risks and therapeutic potential of combined BTK and PI3Kδ inhibition.

## Perspective: Targeting PI3K in Other B Cell Cancers

In this chapter, we have focused on PI3Kδ signaling in normal B cells and the relevance of this to the treatment of CLL with the recently approved drug idelalisib—the first PI3K inhibitor to be approved for clinical use. Idelalisib is also licensed for treatment of indolent non-Hodgkins lymphoma, and with time other cancers may also be found to respond. It is worth noting, for instance, that certain subtypes of DLBCL have a BCR activation profile and it has been suggested that these may benefit from PI3K inhibition. It has also been shown recently that a subset of acute lymphoblastic leukemia may benefit from PI3K inhibition (Geng et al. 2015). However, it is possible that immature B cell leukemia may require dual inhibition of PI3Kδ and PI3Kα, reflecting the redundant role of these isoforms during early B cell development and tonic BCR signaling (Ramadani et al. 2010). It has also been suggested that dual PI3Kα and PI3Kδ inhibition may be required for inhibition in mantle cell lymphoma (Iyengar et al. 2013).
